# Cell-Mediated Immunity (CMI) for SARS-CoV-2 Infection Among the General Population of North India: A Cross-Sectional Analysis From a Sub-sample of a Large Sero-Epidemiological Study

**DOI:** 10.7759/cureus.48824

**Published:** 2023-11-15

**Authors:** Puneet Misra, Pramod K Garg, Amit Awasthi, Shashi Kant, Sanjay K Rai, Mohammad Ahmad, Randeep Guleria, Trideep J Deori, Suprakash Mandal, Abhishek Jaiswal, Gaurav Gongal, Siddhesh Vishwakarma, Mohan Bairwa, Rakesh Kumar, Partha Haldar, Akshay Binayke

**Affiliations:** 1 Epidemiology and Public Health, Centre for Community Medicine, All India Institute of Medical Sciences, New Delhi, New Delhi, IND; 2 Gastroenterology, All India Institute of Medical Sciences, New Delhi, New Delhi, IND; 3 Allergy and Immunology, Centre for Immunobiology and Immunotherapy, Translational Health Science and Technology Institute, Faridabad, IND; 4 Epidemiology and Public Health, World Health Organization, New Delhi, IND; 5 Pulmonary, Critical Care, and Sleep Medicine, All India Institute of Medical Sciences, New Delhi, New Delhi, IND; 6 Community Medicine, All India Institute of Medical Sciences, New Delhi, New Delhi, IND; 7 Allergy and Immunology, Immunology Core Laboratory, Translational Health Science and Technology Institute, Faridabad, IND; 8 Preventive Medicine, Centre for Community Medicine, All India Institute of Medical Sciences, New Delhi, New Delhi, IND

**Keywords:** ifn-γ response, t-cell mediated immunity, covid-19, cell mediated immunity, sars-cov-2

## Abstract

Background

Cell-mediated immunity (CMI), or specifically T-cell-mediated immunity, is proven to remain largely preserved against the variants of severe acute respiratory syndrome coronavirus 2 (SARS-CoV-2), including Omicron. The persistence of cell-mediated immune response in individuals longitudinally followed up for an extended period remains largely unelucidated. To address this, the current study was planned to study whether the effect of cell-mediated immunity persists after an extended period of convalescence or vaccination.

Methods

Whole blood specimens of 150 selected participants were collected and tested for Anti-SARS-CoV-2 Interferon-gamma (IFN-γ) response. Ex vivo SARS-CoV-2-specific interferon-gamma Enzyme-linked Immunospot (IFN-γ ELISpot) assay was carried out to determine the levels of virus-specific IFN-γ producing cells in individual samples.

Findings

Out of all the samples tested for anti-SARS-CoV-2 T-cell-mediated IFN-γ response, 78.4% of samples were positive. The median (interquartile range) spots forming units (SFU) per million levels of SARS-CoV-2-specific IFN-γ producing cells of the vaccinated and diagnosed participants was 336 (138-474) while those who were vaccinated but did not have the disease diagnosis was 18 (0-102); the difference between the groups was statistically significant. Since almost all the participants were vaccinated, a similar pattern of significance was observed when the diagnosed and the never-diagnosed participants were compared, irrespective of their vaccination status.

Interpretations

Cell-mediated immunity against SARS-CoV-2 persisted, irrespective of age and sex of the participant, for more than six months of previous exposure. Participants who had a history of diagnosed COVID-19 infection had better T-cell response compared to those who had never been diagnosed, in spite of being vaccinated.

## Introduction

On the 30th of January 2020, the World Health Organization (WHO) announced that COVID-19 was a public health emergency of international concern. There still persist many questions regarding the key epidemiological and serologic characteristics of the novel pathogen, questions particularly pertaining to its transmissibility (i.e., ability to spread in a population) and its virulence (i.e. case severity) [1]. As of 7 May 2023, worldwide over 765 million confirmed cases and over 6·9 million deaths have been reported owing to COVID-19 infection [2]. However, these case counts certainly underestimate the true cumulative incidence of infection [3] because of the unavailability of diagnostic tests [4], barriers to testing accessibility [5], and asymptomatic infections [6].

Hence, seroprevalence studies are required to get refined estimates of the extent of infection, particularly through population-based serological surveys [7]. These surveys can also provide an estimate of the proportion of the population still susceptible to the infection since antibodies are considered to be a proxy of immunity. Additionally, as the world moves through to an era of vaccine and virus variant, synthesizing sero-epidemiological findings are increasingly vital in tracking the spread of infection, identifying the disproportionately affected groups, and measuring the progress towards herd immunity [1].

However, the presence of anti-SARS-CoV-2 IgG does not necessarily imply protection against COVID-19 infection. It has been documented that despite the presence of anti-spike IgG, the functional neutralizing antibodies against SARS-CoV-2 were observed in only about 70% of the individuals [8,9]. Besides, it has also been observed that the humoral antibodies wane over time, exhibiting a significant decline in antibody titers months after antigenic exposure [10-13].

The emergence of highly immune evasive sub-variants, such as Omicron, has led to the ineffectiveness of antibodies induced by the ancestral WA1 wild-type strain of SARS-CoV-2 in neutralizing Omicron and its sub-variants, including BQ.1, BQ.1.1, XBB, and XBB.1 [9,14,15]. Neutralization by sera from convalescent and vaccinated individuals has been markedly impaired, with titers being lowered by up to 155 fold, even in individuals who received a booster vaccination with a WA1/BA.5 bivalent mRNA vaccine [16]. This highlights the challenges posed by the evolving virus variants and the waning effectiveness of antibodies over time.

However, despite the ineffective antibody neutralization against SARS-CoV-2 variants and the decline in antibody levels, clinical data indicates that hospitalization and severe illness remain relatively uncommon [17]. This suggests the involvement of another arm of the adaptive immune system in providing protection.

Cell-mediated immunity (CMI), specifically T-cell mediated immunity, has been shown to remain largely preserved against SARS-CoV-2 variants, including Omicron [18,19]. Furthermore, the longevity of virus-specific T-cell response has been demonstrated by the detection of SARS-CoV-1 N-reactive CD4 and CD8 memory T cells in individuals who had recovered from SARS 17 years ago in 2003 [20]. The persistence of cell-mediated immune response in individuals longitudinally followed up for an extended period, such as more than six months post-vaccination or infection, is still not well understood.

To address this gap in knowledge, the current study was designed to utilize a subset of 150 individuals from an existing cohort of 10,000 participants under the WHO Unity protocol [21,22]. The aim was to investigate whether the effect of cell-mediated immunity persists after an extended period of convalescence or vaccination. Understanding the durability of cell-mediated immune response is crucial in assessing the long-lasting protection it may provide.

There remains an urgent need to comprehend the immune response that occurs following SARS-CoV-2 infection. This understanding is crucial for determining the significance of the immune response in the course of the disease and especially its potential for providing long-lasting protection. Continued research in this area is vital for informing public health strategies, vaccine development, and understanding the dynamics of immune responses against SARS-CoV-2.

Our primary objective revolved around assessing the duration of acquired immunity and examining the factors that contribute to a suboptimal humoral immune response against SARS-CoV-2. Moreover, we are currently engaged in evaluating the duration and dynamics of cellular immune responses to SARS-CoV-2 variants in individuals who have been vaccinated or have recovered from the infection.

## Materials and methods

Objective

The objectives of the study are: (a) to assess the cell-mediated immunity (CMI) response among the study participants, and (b) to compare the cellular immunity among SARS-CoV-2 antibody-positive and negative participants; and among symptomatic and asymptomatic participants of past COVID-19 infection.

Study design

**Figure 1:**
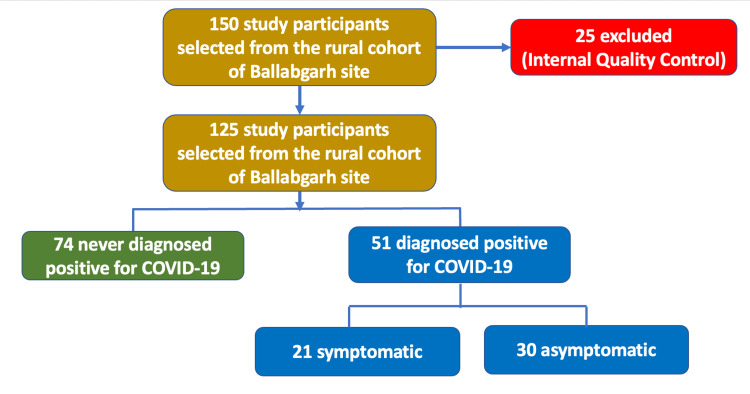
Algorithm for the selection of study participants

The current study was part of a larger study under the WHO Unity protocol. A cohort of 10,000 individuals was assembled for a population-based, age-stratified sero-epidemiological study for COVID-19 virus infection from five selected states in India covering rural, urban, and tribal areas. As the information on the cell-mediated immune response to COVID-19 was limited, and due to logistic and operational reasons, it was planned to assess the cell-mediated immunity only in a subset of the original cohort from one selected site (All India Institute of Medical Sciences, New Delhi). A subset of 150 individuals was taken from the selected study site and blood specimens were collected between April-June 2022. Initially, it was planned to collect blood specimens from individuals based on their vaccination status. However, by the time data collection started, it was seen that almost all individuals in the study cohort were vaccinated. The study participants were finally selected according to the flowchart in Figure 1.

Participant characteristics

Whole blood specimens of 150 selected participants were collected and tested for anti-SARS-CoV-2 interferon-gamma (IFN-γ) response. However, only 125 samples were considered for statistical analysis since 25 samples were excluded due to internal quality control criteria. The participants consisted of two categories. Firstly, those with documented positive cases, labelled as “diagnosed”, and tested either by real-time reverse transcription-polymerase chain reaction (RT-PCR) or rapid antigen tests (RAT) (n=51 out of 125 total participants). The second category labelled as “never diagnosed” were participants who had never been diagnosed with SARS-CoV-2 viral infection and also whose exposure status was unknown (n=74 out of 125 total participants). The blood specimens were collected after ensuring that six or more months had elapsed since convalescence and/or vaccination.

Peripheral blood mononuclear cell (PBMC) isolation and preparation

Approximately eight milliliters of whole blood specimen was collected from the participants. Peripheral blood mononuclear cells (PBMCs) were isolated from whole blood by using the ficoll-histopaque (Lymphoprep, SerumWerk, Bernburg, Germany)-based density gradient centrifugation method [23]. Post isolation, the PBMCs were washed twice with sterile 1X phosphate buffered saline (PBS) and cryopreserved using a freezing media containing 90% Fetal Bovine Serum (FBS) (Gibco, Thermofisher Scientific, Waltham, USA) + 10% dimethyl sulfoxide (DMSO) (Merck, Sigma, Burlington, USA) and stored in liquid nitrogen until further use.

Subsequently, the cryopreserved PBMCs were thawed by directly warming them at 37°C in a water bath and washed using sterile pre-warmed complete RPMI 1640 medium supplemented with 10% FBS (containing L-glutamine, HEPES, 1% penicillin: streptomycin solution (Thermofisher Scientific, Waltham, USA), 1X MEM Vitamin (Thermofisher Scientific, Waltham, USA), 5 mM sodium pyruvate (Gibco), 1X MEM Non-essential Amino acids (Gibco); all the components were filtered using 0·2 µm sterile filter]. The PBMCs were rested for two hours in complete RPMI media supplemented with benzonase (Merck, Sigma) at 37°C before enumeration using haemocytometer.

Interferon-gamma enzyme-linked immunospot (IFN-γ ELISpot) assay

Ex-vivo SARS-CoV-2 specific IFN-γ ELISpot (MabTech Human IFN-γ ELISpotPLUS kit (ALP), Nacka Strand, Sweden) was carried out to determine the levels of virus-specific IFN-γ producing cells in individual samples as described previously [24]. The pre-coated wells in the ELISpot plate were conditioned using sterile complete RPMI 1640 medium supplemented with 10% FBS and then incubated for 18-20 hrs at 37°C and 5% CO2 with the 0.25 million PBMCs/well and appropriate stimulants. To test the T-cell response, the PBMCs were stimulated with the virus-specific pool of immunodominant human leukocyte antigen (HLA) class I & II-restricted T-cell epitopes of SARS-CoV-2 proteome (PanSARS-CoV-2 PepMix peptide pool, JPT Peptide Technologies, Berlin, Germany) at a concentration of 1 µg/ml of each peptide. As an internal negative control, each PBMC sample was also stimulated with an equimolar concentration of DMSO, whereas for positive control, PBMCs were stimulated with 5 µg/ml anti-CD3 antibody. Co-stimulants anti-human CD28 and anti-human CD49d at a final concentration of 1 µg/ml were added in the test and negative control wells. Post incubation, the plate was developed as per the manufacturer’s guidelines, and the spots were quantified using a CTL Fluorescence S6 Universal reader (CTL, Cleveland, USA).

Human ethics

The PBMCs were isolated from the samples which were collected from healthy convalescent/vaccinated participants after obtaining formal written informed consent. Institutional Ethical approval (IEC-959/04.09.2020) for the study protocol was taken from both the participating institutes (All India Institute of Medical Sciences, New Delhi and Translational Health Science and Technology Institute, Faridabad).

Data analysis

The socio-demographic information of the participants was exported to Microsoft Excel software (Microsoft Corporation, Redmond, USA), and data analysis was conducted using the statistical software STATA Version 12 (STATA Corporation, College Station, USA). A qualified data manager and the study investigators collaborated to perform data cleaning using both Microsoft Excel and STATA. Descriptive statistical analysis was carried out, and the results were presented as proportions for categorical variables and as mean (SD) with a 95% confidence interval (CI) for continuous variables. The seroprevalence was reported as a percentage with a 95% CI, categorized according to the study site, round, urban-rural area, age group, sex, presence of symptoms, and vaccination status. The adjusted prevalence with 95% CI was calculated after correcting for the test accuracy.

For the ELISpot data, the number of spots forming units (SFUs) per million PBMCs was calculated by multiplying the background subtracted spots per well by four. Negative values were set to zero. Samples with a low anti-CD3 response (<45 SFUs/million PBMCs) were excluded from the analysis.

To compare the levels of SARS-CoV-2-specific IFN-γ producing cells (SFUs/million PBMCs) between the study groups, the Mann-Whitney U test was employed. A significance level of p<0·05 was deemed statistically significant. The statistical analysis and graphical representations of the data were conducted using Graphpad Prism 8 software (Graphpad, San Diego, USA).

## Results

In this study, a cohort of 150 healthy participants was initially enrolled. Following the application of internal quality control criteria, 25 participants were excluded, resulting in a final sample size of 125 participants for analysis (Table 1). The level of cell-mediated immunity was assessed using the ELISpot assay, which allowed for the quantification of anti-SARS-CoV-2-specific IFN-γ-producing T cells in terms of spot-forming units per million PBMCs.

**Table 1:** Distribution of participants by selected variables *Excluding participants who took the vaccine, but the type is unknown **Symptoms include vomiting, nausea, rashes, conjunctivitis, muscle ache, joint ache, loss of appetite/smell/taste, nose bleeding, fatigue, seizures, and other neurological symptoms

	Males (n = 65)	Females (n = 60)	Total (n = 125)
Age			
14-18y	2	2	4
19-60y	49	49	98
>60y	14	9	23
Unvaccinated	1	-	1
Vaccinated	64	60	124
Covaxin	37*	35*	72
Covishield	24*	23*	47
Documented positive for SARS-CoV-2	30	21	51
Symptomatic	13	8	21
Asymptomatic	17	13	30
Symptoms			
Fever	5	4	9
Sore throat	2	1	3
Cough	3	1	4
Headache	1	1	2
Others**	1	2	3
Never Diagnosed for SARS-CoV-2	35	39	74

The ages of the 125 participants ranged from 14 years to 80 years, which were subdivided into three age groups: 14-18 years (n=4), 19-60 years (n=98), and >60 years (n=23). Almost equal proportions of both genders were recruited (52% males and 48% females). Of the 125, 40.8% (n=51) individuals had laboratory confirmation for SARS-CoV-2 diagnosis using either RT-PCR or RAT, hence called “dignosed,” and the rest were “never diagnosed” (Table 2). Among the diagnosed participants, 58.8% (n=30/51) had asymptomatic infection with SARS-CoV-2, and 41.17% (n=21/51) had symptomatic infections with symptoms including fever, sore throat, cough, headache, and others (Table 1).

Nearly all of the participants (99.2%, n=124/125) in the study had received vaccination. The majority of them (57.6%, n=72/124) were vaccinated with Covaxin (BBV152), while a significant portion (37.6%, n=47/124) received Covishield (ChAdOx nCoV-19). Among the vaccinated participants, 4.83% (n=6/124) received a single dose, while the remaining 95.16% (n=118/124) received the recommended double dose. It is worth noting that four participants had received vaccination but could not recall the specific type of vaccine they received. One participant reported receiving the Sputnik V vaccine, while only one participant remained unvaccinated throughout the study. This vaccination distribution reflects the diverse vaccination status within the participant pool, which is essential for examining the relationship between vaccination and immune response.

Out of the 125 PBMC samples tested for anti-SARS-CoV-2 T cell-mediated IFN-γ response, 78.4% (n=98/125) samples were positive. The median (IQR) SARS-CoV-2-specific IFN-γ SFU/million cells were comparable across the different age groups (209 (22.5-360.5) vs 77 (4 - 371.5) vs 88 (0-220) for age groups 14-18 years, 19-60 years and >60 years respectively). The median of SARS-CoV-2 specific T cells was higher in participants aged 14-18 years when compared to other age groups but the difference was statistically non-significant. The million cells SFU were compared among males and females and the median (IQR) was 118 (18-383) and 48 (0-289), respectively (Table 2).

**Table 2:** The SARS-CoV-2 specific IFN-γ levels among participants by age group and sex *ns=nonsignificant, p-value>0·05 considered as significant, SFU=spot-forming units, IQR=interquartile range

	SFU/million cells (median with IQR)	P-value*			
		14-18y vs 19-60y	19-60y vs >60y	14-18y vs >60y	Gender
Age		ns	ns	ns	-
14-18y (n=4)	209 (22.5 - 360.5)				
19-60y (n=98)	77 (4 - 371.5)				
>60y (n=23)	88 (0 - 220)				
Sex		-	-	-	ns
Male (n=65)	118 (18 - 383)				
Female (n=60)	48 (0 - 289)				
Total participants	n=125				

**Figure 2:**
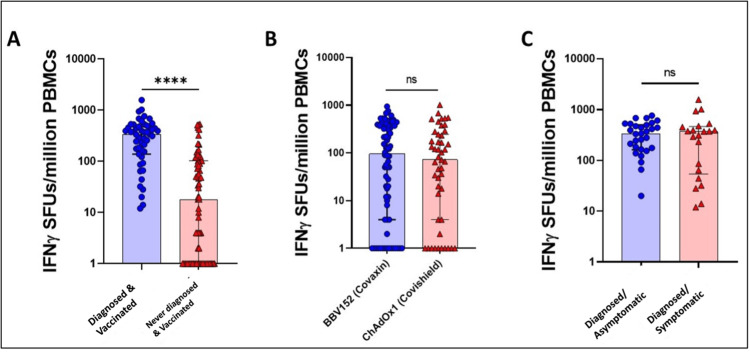
The level SARS-CoV-2 antigen-specific T cells in various convalescent and vaccinated individuals six months since last exposure ns=non-significant; **** p-value<0·0001 IFN-γ SFUs/million PBMCs=interferon-gamma spot-forming unit per million peripheral blood mononuclear cells

Since, almost all the participants were vaccinated, similar pattern of significance was observed when diagnosed and never-diagnosed participants were compared, irrespective of their vaccination status (Median (IQR) SFU/million: 336 (138-474) vs 16 (0-102) for diagnosed and undiagnosed respectively (Figure 1)). Among the vaccinated group, irrespective of prior infection, the comparison of IFN-γ levels among those who received Covaxin (BBV152) and those who received Covishield (ChAdOx1) showed no statistical difference (p-value 0.6099, Median (IQR) SFU/million: 96 (4-373) vs 72 (4-244) for Covaxin and Covishield respectively (Table 3, Figure 2B)). No significant difference was observed when IFN-γ levels were compared among participants who recovered from asymptomatic or symptomatic SARS-CoV-2 infection (Median (IQR) SFU/million: 334 (161.5-501) vs 370 (54-463), respectively (Table 3, Figure 2C)).

**Table 3:** Median (IQR) level of SARS-CoV-2 specific IFN-γ levels among participants with selected characteristics *ns=nonsignificant, p-value<0·05 considered as significant, ^a^irrespective of the vaccination status, ^b^irrespective of the exposure to SARS-Cov-2, SFU=Spot-forming units, IQR=interquartile range

	Male/Female (n)	SFU/million cells (median with IQR)	p-value*
Diagnosed + Vaccinated (n=51)	30/21	336 (138 - 474)	<0·0001
Never diagnosed+ Vaccinated (n=73)	34/39	18 (0 - 102)	
Diagnosed^a^ (n=51)	30/21	336 (138 - 474)	<0·0001
Never diagnosed^a^ (n=74)	35/39	16 (0 - 102)	
Diagnosed – Asymptomatic (n=30)	17/13	334 (161.5 - 501)	ns
Diagnosed – Symptomatic (n=21)	13/8	370 (54 - 463)	
Vaccinated (Covaxin)^b ^(n=72)	37/35	96 (4 - 373)	ns
Vaccinated (Covishield)^b^ (n=47)	24/23	72 (4 - 244)	
Total Participants	n=125		

Within the vaccinated group, irrespective of prior infection, no statistical difference in IFN-γ levels was found when comparing individuals who received Covaxin (BBV152) to those who received Covishield (ChAdOx1) (p-value=0.6099, Median (IQR) SFU/million: 96 (4-373) vs 72 (4-244), respectively (Table 3, Figure 2B)). This implies that both vaccines elicit comparable T-cell responses in terms of IFN-γ production.

Furthermore, when comparing IFN-γ levels between participants who had recovered from asymptomatic and symptomatic SARS-CoV-2 infection, no significant difference was observed (Median (IQR) SFU/million: 334 (161.5-501) vs 370 (54-463), respectively). The presence of symptoms during the infection does not significantly impact the magnitude of the T-cell immune response (Table 3, Figure 2C).

Overall, these results highlight the importance of both prior infection and vaccination in enhancing the cellular immune response, as indicated by higher IFN-γ levels. Moreover, they demonstrate the similar T-cell responses generated by Covaxin and Covishield vaccines, irrespective of prior infection. Additionally, the absence of a significant difference in IFN-γ levels between asymptomatic and symptomatic individuals suggests that the severity of symptoms may not be a major determinant of T-cell immune response.

The frequency of SARS-CoV-2-specific IFN-γ spot-forming T cells was compared among multiple study groups. The T-cell immune response was compared based on (a) Diagnosis with SARS-CoV-2 infection in vaccinated individuals, (b) Type of vaccination, and (c) Asymptomatic or symptomatic infection of SARS-CoV-2 in diagnosed indviduals. The y-axis represents the IFN-γ SFUs per million PBMCs stimulated overnight with the SARS-CoV-2 peptide pool. A statistically significant difference was observed between the diagnosed and never-diagnosed participants who had been vaccinated. No statistically significant difference was seen in the other two comparative groups (Figure 2).

**Figure 3:**
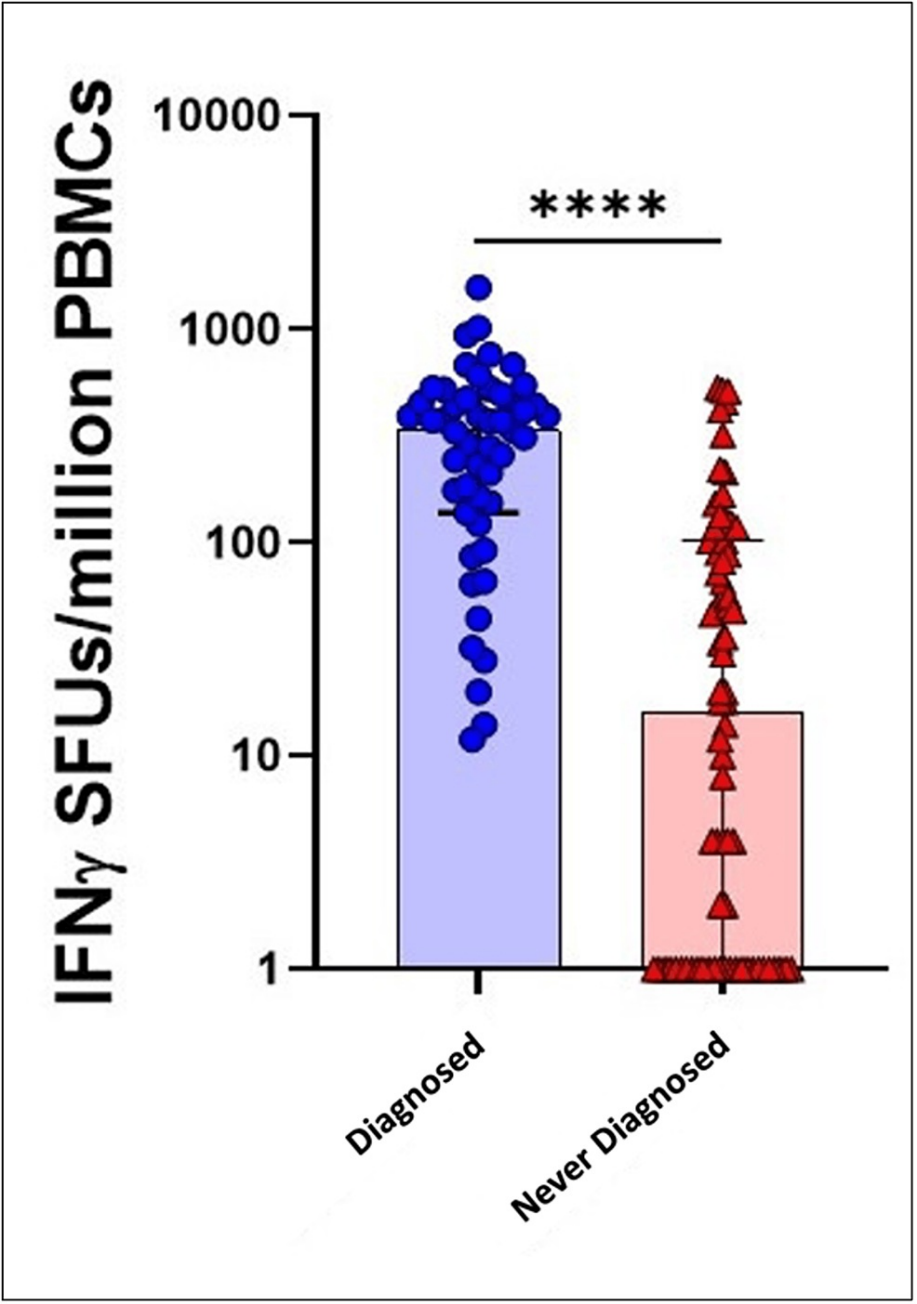
SARS-CoV-2 IFN-γ levels in diagnosed and never-diagnosed participants irrespective of vaccination status Comparison of the levels of SARS-CoV-2 specific IFN-γ producing cells among participants with pre-exposure to SARS-CoV-2 and those who were never diagnosed with SARS-CoV-2, irrespective of their vaccination status. ****p-value<0.0001 (statistically highly significant) IFN-γ SFUs/million PBMCs=interferon-gamma spot-forming unit per million peripheral blood mononuclear cells

When comparing participants who had been diagnosed with COVID-19 to those who had never been diagnosed, regardless of their vaccination status, a similar pattern of significance was observed. The levels of IFN-γ producing cells were significantly higher in diagnosed participants compared to undiagnosed participants (Median (IQR) SFU/million: 336 (138-474) vs 16 (0-102), respectively (Figure 3)). This finding suggests that prior infection plays a crucial role in enhancing cellular immune response, regardless of vaccination status.

## Discussion

The persistence of T-cell mediated immunity for an extended duration, post-recovery is well documented in SARS-CoV-2 [25,26]. However, only a few studies are available on the persistence of the SARS-CoV-2-specific T-cell immune response along with its correlation with demographic characteristics like age, sex, type of infection, and type of vaccination. We tried to fill this gap through the current study. 150 selected participants were enrolled in the study out of which 125 samples were considered for statistical analysis since 25 samples were excluded due to internal quality control criteria. The participants were selected from an already existing 10,000 individuals being followed up under the WHO Unity protocol seroepidemiological study. In this study, the level and longevity of SARS-CoV-2-specific T-cell mediated IFN-γ response was determined using the ELISpot assay. We included individuals where more than six months had elapsed since infection and/or vaccination. We assessed the association between the persistence of T-cell immunity and age groups, sex, the type of vaccination, and symptoms when exposed to SARS-CoV-2.

Sette and Crotty (2021) reported that elderly individuals possessed a smaller naïve T-cell population and were also susceptible to immunosenescence contributing to the ineffective response against SARS-CoV-2 infection [27,28]. However, Peluso et al. (2021) reported that participants older than 50 years of age had a higher percentage of SARS-CoV-2 N- and S-specific IFNγ+ CD4+ T cells four months post onset of illness measured by intracellular cytokine staining [29]. Arankalle et al. (2022) found that participants older than 55 years exhibited robust T-cell responses to the whole virion-inactivated vaccine like Covaxin (BBV152) measured by ELISpot [30,31]. We found that higher levels of IFN-γ producing cells among participants of the age group 14-18 years. However, this difference was not statistically significant across the multiple age groups studied (14-18y vs 19-60y vs >60y). One possible explanation for our finding could be the overall small number of participants, particularly in the age group 14-18 years (N=4). Therefore, we might have missed the difference, even if it truly existed. However, our finding is in agreement with a study by Yan et al. (2022), where IFN-γ persisted independent of age [25,26].

Takahashi et al. (2020) in a study among 98 hospital-admitted participants with confirmed COVID-19 diagnosis reported that females possessed more robust T-cell activation than male patients. In addition, the disease outcome was worse in males than in female patients [32]. Scully et al. (2020) reported that the case-fatality rate among males across 38 countries was 1.7 times higher than the average female case-fatality rate [33]. Although there were notable differences in the immune response between males and females during an active SARS-CoV-2 infection, Killic et al. (2021) observed that the levels of IFN-γ production remained similar between the two genders, suggesting that a higher number of T-cells does not necessarily result in increased IFN-γ release [34]. Similarly, in our study, we observed comparable levels of SARS-CoV-2-specific IFN-γ among males and females. However, we did find that males exhibited higher levels of spot-forming units (SFUs) compared to females, indicating a potentially greater T-cell response in males.

Convalescent phase SARS-CoV-2-specific CD4+ and CD8+ cells pre-dominantly express IFN-γ [17,27]. The persistence of such T-cells is well-documented in studies by Peluso et al. (2021) and Sherina et al. (2021). They employed activation-induced marker assay/intracellular cytokine staining assays [29,35]. The SARS-CoV-2-specific T cells persist for 17-18 months after the onset of the illness [26]. Of the 125 convalescent participants of our study, 51 had documented previous exposure to SARS-CoV-2, 6-12 months prior to the date of sample collection. We used the ELISpot method and detected robust and significantly higher SARS-CoV-2-specific T-cell IFN-γ response in the diagnosed cohort compared to those who did not have previous documented exposure, irrespective of their vaccination status.

All the diagnosed and undiagnosed participants in the study were vaccinated except one participant in the never-diagnosed category who was unvaccinated. A marked difference was observed in the levels of IFN-γ producing cells between the participants of the diagnosed-vaccinated category and the never-diagnosed-vaccinated category. This observation suggests that the T-cell immunity generated over the period of 6-12 months post-onset of illness is better among individuals who had been previously exposed to SARS-CoV-2 infection, irrespective of vaccination status. A similar finding has also been reported by Reynolds et al. (2021). They reported that at 42 weeks, enhanced T-cell immunity developed against SARS-CoV-2 spike protein in healthcare workers (HCWs) who were vaccinated and had previous exposure to infection compared to those who were only vaccinated [36,37]. Another study by Zuo et al. (2021) using ELISpot and Intracellular cytokine staining on 100 convalescent donors 6 months post SARS-CoV-2 infection found that the T-cell response was higher in donors who had experienced a symptomatic infection from SARS-CoV-2 [25].

We compared the T-cell immunity among the diagnosed participants on the basis of symptomatic and asymptomatic infection, >6 months back. The T-cell immunity was comparable but slightly higher in those who had symptomatic infection. Our finding was thus comparable with previously reported findings [25,27].

Arankalle et al. (2022), reported that covaxin (BBV152) elicited a significantly superior IFN-γ T-cell response compared to Covishield (ChAdOx1 nCoV- 19) in a study containing a cohort of 187 Covishield and 21 Covaxin recipients [30]. We employed the same method and our cohort of Covaxin was larger compared to Covishield. We did not find any significant difference in the levels of SARS-CoV-2 specific IFN-γ producing cells between the two groups.

Limitations

The current study had several limitations that should be acknowledged. Firstly, the sample size was relatively small, with only 150 participants selected from an original cohort of 10,000 individuals. Additionally, 25 participants had to be excluded due to internal quality control issues, further reducing the effective sample size. Consequently, the findings should be interpreted with caution, and the generalizability of the results may be limited.

Another limitation of the study was the utilization of the ELISpot method to determine the SARS-CoV-2 memory response by measuring total IFN-γ producing cells. This approach can be considered a drawback since it did not allow for the specific characterization of CD4+ and CD8+ T-cell responses. An in-depth analysis of these T-cell subsets could provide a more comprehensive understanding of the immune response to SARS-CoV-2.

Despite these limitations, the present study contributes valuable insights into the immune response against SARS-CoV-2. Future research with larger sample sizes and more extensive methodologies, such as flow cytometry, could provide a more detailed characterization of T-cell subsets and improve our understanding of the immune response dynamics.

It is crucial to address these limitations in future studies to further elucidate the intricacies of the immune response to SARS-CoV-2 and obtain a more comprehensive picture of the protective mechanisms involved. Nonetheless, the current study provides initial evidence and paves the way for further investigations in this field.

## Conclusions

The present study indicates that irrespective of age and sex of the participant, T-cell immune response against SARS-CoV-2 was observed for more than six months of previous exposure either through vaccination or natural infection. Over three-quarters of the participants demonstrated a CMI response characterized by the production of IFN-γ. Individuals who had experienced natural infection displayed a more robust CMI response, irrespective of their vaccination status. 

This underscores the importance of a history of diagnosed COVID-19 infection in enhancing T-cell response compared to individuals who had never been diagnosed, regardless of their vaccination status. This suggests that prior exposure through natural infection confers a certain level of immunological advantage in terms of CMI.

The persistence of CMI suggests that vaccinated individuals and those who have recovered from previous infections may have a continued defence against the virus and potentially a reduced risk of reinfection.

## References

[1] Bobrovitz N, Arora RK, Cao C (2021). Global seroprevalence of SARS-CoV-2 antibodies: a systematic review and meta-analysis. PLoS One.

[2] (2023). Weekly epidemiological update on COVID-19 - 21 December 2021 | World Health Organization. https://www.who.int/publications/m/item/weekly-epidemiological-update-on-covid-19---21-december-2021#:~:text=Weekly%20epidemiological%20update%20on%20COVID%2D19%20%2D%2021%20December%202021,-Edition%2071&text=Nonetheless%2C%20this%20corresponds%20to%20over,deaths%20have%20been%20reported%20globally..

[3] Cheng MP, Papenburg J, Desjardins M (2020). Diagnostic testing for severe acute respiratory syndrome-related coronavirus 2: a narrative review. Ann Intern Med.

[4] (2022). Supply Shortages Impacting COVID-19 and Non-COVID Testing | American Society for Microbiology. https://asm.org/Articles/2020/September/Clinical-Microbiology-Supply-Shortage-Collecti-1.

[5] Lieberman-Cribbin W, Tuminello S, Flores RM, Taioli E (2020). Disparities in COVID-19 testing and positivity in New York City. Am J Prev Med.

[6] Buitrago-Garcia D, Egli-Gany D, Counotte MJ (2020). Occurrence and transmission potential of asymptomatic and presymptomatic SARS-CoV-2 infections: a living systematic review and meta-analysis. PLoS Med.

[7] Grant R, Dub T, Andrianou X, Nohynek H, Wilder-Smith A, Pezzotti P, Fontanet A (2021). SARS-CoV-2 population-based seroprevalence studies in Europe: a scoping review. BMJ Open.

[8] Misra P, Kant S, Guleria R (2022). Antibody response to SARS-CoV-2 among COVID-19 confirmed cases and correlates with neutralizing assay in a subgroup of patients in Delhi National Capital Region, India. Vaccines (Basel).

[9] Linderman SL, Lai L, Bocangel Gamarra EL (2022). Neutralizing antibody responses in patients hospitalized with SARS-CoV-2 Delta or Omicron infection. J Clin Invest.

[10] Lau EH, Tsang OT, Hui DS (2021). Neutralizing antibody titres in SARS-CoV-2 infections. Nat Commun.

[11] Cohen KW, Linderman SL, Moodie Z (2021). Longitudinal analysis shows durable and broad immune memory after SARS-CoV-2 infection with persisting antibody responses and memory B and T cells. Cell Rep Med.

[12] GeurtsvanKessel CH, Geers D, Schmitz KS (2022). Divergent SARS-CoV-2 Omicron-reactive T and B cell responses in COVID-19 vaccine recipients. Sci Immunol.

[13] Hoffmann M, Krüger N, Schulz S (2022). The Omicron variant is highly resistant against antibody-mediated neutralization: implications for control of the COVID-19 pandemic. Cell.

[14] Garcia-Beltran WF, St Denis KJ, Hoelzemer A (2022). mRNA-based COVID-19 vaccine boosters induce neutralizing immunity against SARS-CoV-2 Omicron variant. Cell.

[15] Dejnirattisai W, Huo J, Zhou D (2022). SARS-CoV-2 Omicron-B.1.1.529 leads to widespread escape from neutralizing antibody responses. Cell.

[16] Wang Q, Iketani S, Li Z (2023). Alarming antibody evasion properties of rising SARS-CoV-2 BQ and XBB subvariants. Cell.

[17] Sekine T, Perez-Potti A, Rivera-Ballesteros O (2020). Robust T cell immunity in convalescent individuals with asymptomatic or mild COVID-19. Cell.

[18] Tarke A, Coelho CH, Zhang Z (2022). SARS-CoV-2 vaccination induces immunological T cell memory able to cross-recognize variants from Alpha to Omicron. Cell.

[19] Gao Y, Cai C, Grifoni A (2022). Ancestral SARS-CoV-2-specific T cells cross-recognize the Omicron variant. Nat Med.

[20] Le Bert N, Tan AT, Kunasegaran K (2020). SARS-CoV-2-specific T cell immunity in cases of COVID-19 and SARS, and uninfected controls. Nature.

[21] Misra P, Kant S, Guleria R (2022). Serological prevalence of SARS-CoV-2 antibody among children and young age group (between 2 and 17 years) in India: an interim result from a large multicentric population-based seroepidemiological study. J Family Med Prim Care.

[22] (202321). Population-based age-stratified seroepidemiological investigation protocol for coronavirus 2019 (COVID-19) infection [Internet]. [cited. https://www.who.int/publications-detail-redirect/WHO-2019-nCoV-Seroepidemiology-2020.2.

[23] Binayke A, Zaheer A, Dandotiya J (2022). Proinflammatory innate cytokines and distinct metabolomic signatures shape the T Cell response in active COVID-19. Vaccines (Basel).

[24] Thiruvengadam R, Awasthi A, Medigeshi G (2022). Effectiveness of ChAdOx1 nCoV-19 vaccine against SARS-CoV-2 infection during the delta (B.1.617.2) variant surge in India: a test-negative, case-control study and a mechanistic study of post-vaccination immune responses. Lancet Infect Dis.

[25] Zuo J, Dowell AC, Pearce H (2021). Robust SARS-CoV-2-specific T cell immunity is maintained at 6 months following primary infection. Nat Immunol.

[26] Yan LN, Liu PP, Li XG (2021). Neutralizing antibodies and cellular immune responses against SARS-CoV-2 sustained one and a half years after natural infection. Front Microbiol.

[27] Sette A, Crotty S (2021). Adaptive immunity to SARS-CoV-2 and COVID-19. Cell.

[28] Chen Z, John Wherry E (2020). T cell responses in patients with COVID-19. Nat Rev Immunol.

[29] Peluso MJ, Deitchman AN, Torres L (2021). Long-term SARS-CoV-2-specific immune and inflammatory responses in individuals recovering from COVID-19 with and without post-acute symptoms. Cell Rep.

[30] Arankalle V, Kulkarni-Munje A, Kulkarni R (2022). Immunogenicity of two COVID-19 vaccines used in India: an observational cohort study in health care workers from a tertiary care hospital. Front Immunol.

[31] Hirai T, Yoshioka Y (2022). Considerations of CD8(+) T cells for optimized vaccine strategies against respiratory viruses. Front Immunol.

[32] Takahashi T, Ellingson MK, Wong P (2020). Sex differences in immune responses that underlie COVID-19 disease outcomes. Nature.

[33] Scully EP, Haverfield J, Ursin RL, Tannenbaum C, Klein SL (2020). Considering how biological sex impacts immune responses and COVID-19 outcomes. Nat Rev Immunol.

[34] Kilic G, Bulut O, Jaeger M (2021). The immunological factors predisposing to severe Covid-19 are already present in healthy elderly and men. Front Immunol.

[35] Sherina N, Piralla A, Du L (2021). Persistence of SARS-CoV-2-specific B and T cell responses in convalescent COVID-19 patients 6-8 months after the infection. Med.

[36] Reynolds CJ, Pade C, Gibbons JM (2021). Prior SARS-CoV-2 infection rescues B and T cell responses to variants after first vaccine dose. Science.

[37] Crotty S (2021). Hybrid immunity. Science.

